# Primary Renal Neuroendocrine Tumor: Diagnostic Challenges in a Rare Entity—A Case Report

**DOI:** 10.3390/curroncol33020105

**Published:** 2026-02-06

**Authors:** Raphaela D. Lewetag, Katharina Kluthe, Nils F. Trautwein, Ulrich M. Lauer, Christian la Fougère, Bence Sipos, Lars Zender, Clemens Hinterleitner, Stephan Singer, Martina Hinterleitner

**Affiliations:** 1 Department of Medical Oncology and Pneumology (Internal Medicine VIII), University Hospital Tuebingen, 72076 Tuebingen, Germany; 2 ENETS Center of Excellence, University Hospital Tuebingen, 72076 Tuebingen, Germanychristian.lafougere@med.uni-tuebingen.de (C.l.F.);; 3 Department of Pathology, University Hospital Tuebingen, 72076 Tuebingen, Germany; 4 Department of Nuclear Medicine and Clinical Molecular Imaging, University Hospital Tuebingen, 72076 Tuebingen, Germany; 5 DFG Cluster of Excellence 2180 ‘Image-Guided and Functional Instructed Tumor Therapy’, University of Tuebingen, 72076 Tuebingen, Germany; 6 German Cancer Consortium, German Cancer Research Center, 72070 Tuebingen, Germany; 7 Cancer Biology and Genetics, Memorial Sloan Kettering Cancer Center, New York, NY 10065, USA

**Keywords:** neuroendocrine neoplasms, primary renal neuroendocrine tumor, immunohistochemistry, tumor genome sequencing, SSTR PET

## Abstract

Neuroendocrine neoplasms (NENs) represent a rare, heterogeneous group of malignancies. Within this tumor entity, primary renal neuroendocrine tumors (PRNETs) are exceedingly rare, with only 160 cases reported worldwide. Due to the absence of native neuroendocrine cells in the renal parenchyma, their cellular origin remains unclear. Clinical and diagnostic challenges are reflected by the low incidence, non-specific clinical presentation, resemblance to common renal neoplasms, and the need for specialized histopathological diagnostic examination. Here, we present the case of a 61-year-old female with an incidentally diagnosed left-sided PRNET in September 2024. This case highlights the diagnostic complexity of PRNET and, importantly, underscores its genomic heterogeneity. It demonstrates a lack of typical renal cell carcinoma (RCC) or common NEN-associated gene alterations, emphasizing their unique molecular landscape.

## Simple Summary

Neuroendocrine neoplasms (NENs) are rare and heterogeneous tumors. Primary renal neuroendocrine tumors (PRNETs) are exceptionally uncommon, with approximately 160 cases reported worldwide. Since PRNETs present with non-specific clinical symptoms and closely mimic more common renal neoplasms, diagnosis is challenging and relies on specialized pathological evaluation. Here, we report the case of a 61-year-old woman with an incidentally discovered left-sided PRNET, illustrating the diagnostic challenges and underscoring the genomic heterogeneity of these tumors.

## 1. Introduction

Neuroendocrine neoplasms (NENs) are a rare and heterogeneous group of solid tumors, accounting for 0.5–2.0% of all newly diagnosed malignancies [1]. They are classified into two groups with distinct biological characteristics: well-differentiated neuroendocrine tumors (NETs) and poorly differentiated neuroendocrine carcinomas (NECs) [2]. These neoplasms can originate from various anatomical sites, most commonly occurring in the gastrointestinal tract, including the pancreas (>60%), and the lungs (>20%) [3]. In contrast to these more prevalent locations, primary renal neuroendocrine tumors (PRNET) are extremely rare [4]. To date, there are approximately 160 cases reported worldwide [5]. Conversely, the cell of origin in PRNET is not finally known, as there are no native neuroendocrine cells in the renal parenchyma described [5]. The current knowledge of this rare entity, as well as its therapeutic regimes, is mainly based on single case reports, small case series, and experts’ recommendations.

Significant diagnostic challenges arise not only due to the low incidence but are also reflected by their unspecific clinical presentation, resembling symptoms observed in other renal diseases such as hematuria and (back) pain. The diagnosis of PRNET is based on histopathological examination, incorporating immunohistochemical markers like chromogranin A and synaptophysin, to confirm neuroendocrine differentiation [6]. Another important diagnostic procedure is functional imaging using Somatostatin Receptor Positron Emission Tomography (SSTR PET).

In this study, we describe a case of a PRNET, consequently outline diagnostic challenges, define genomic characteristics, and perform a critical review of the current literature of this rare entity.

## 2. Case Report

**Figure 1:**
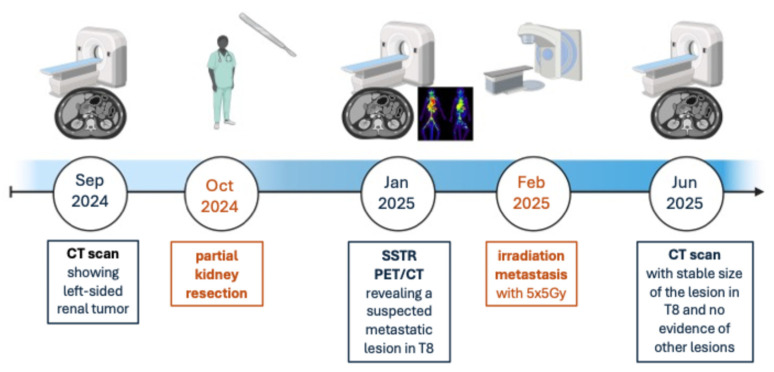
Medical course. Timeline of diagnosis and treatment of the patient with PRNET. Abbreviations: CT = Computed Tomography, Gy = Gray, SSTR PET/CT = Somatostatin Receptor Positron Emission Tomography/Computed Tomography, T8 = Thoracic Vertebra 8. This figure was created using BioRender.com.

In September 2024, a 61-year-old woman was referred for further diagnostic evaluation following the incidental discovery of a renal mass during a routine medical examination (Figure 1). The computed tomography (CT) scan revealed a left-sided renal tumor mass without suspected metastatic lesions. At the time of diagnosis, the patient showed no specific clinical symptoms, specifically denying dysuria, hematuria, B symptoms, as well as neuroendocrine tumor-specific symptoms, including flush and diarrhea. She reported no significant medical history or indicated an increased occurrence of malignancies in her family. In October, the patient underwent a robot-assisted laparoscopy, and a partial kidney resection was performed. As renal cell carcinoma (RCC) was the initial suspected diagnosis, in accordance with current guidelines, primary surgical resection without preoperative biopsy was performed. The tumor was resected in sano and measured 3.7 cm in its greatest dimension with focal infiltration of the perirenal fat corresponding to the TNM classification for renal cell carcinomas (pT3a, L0, V1, pN0, and R0). Histopathological examination revealed an invasively growing epithelial neoplasm with mixed tubular, trabecular, and papillary architecture. The tumor cells showed abundant eosinophilic cytoplasm and centrally located round-to-oval nuclei with finely granular chromatin, consistent with neuroendocrine differentiation. A sharp demarcation from the adjacent renal parenchyma was present.

An extended immunohistochemical panel was applied to address the broad differential diagnosis of renal tumors with neuroendocrine features. The tumor cells were positive for synaptophysin and chromogranin A, confirming neuroendocrine differentiation. CA9 was positive, whereas PAX8 and CK7 were negative, contrasting with conventional renal cell carcinoma subtypes. CD10 showed only focal positivity and, in the absence of supportive morphology and additional renal lineage markers, was not considered indicative of RCC.

To exclude a metastatic neuroendocrine tumor, site-specific markers were assessed. The tumor cells expressed Islet-1, a marker predominantly associated with NEN of pancreatic or rectal origin, but Islet-1 positivity is also described as common in PRNET. Furthermore, Islet-1 expression in this case may support the presence of a well-differentiated neuroendocrine phenotype. The absence of TTF-1 and OTP argued against a pulmonary origin, while negativity for CDX2 excluded a gastrointestinal primary. Digital Ki67 evaluation (using Cognition Master, VMscope GmbH, Berlin, Germany) of areas with the highest Ki67 proliferation (hot spots) accounted for a low Ki-67 proliferation index of 1.6%, consistent with a low-grade neoplasm.

**Figure 2:**
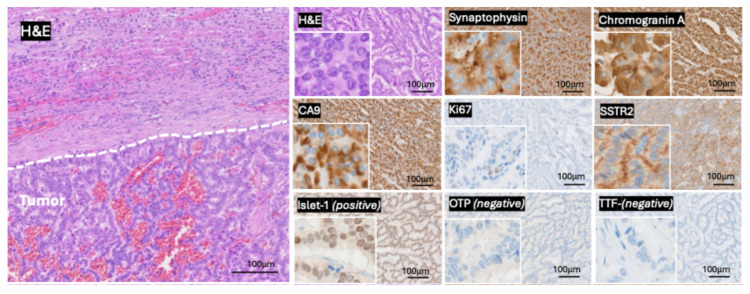
Pathological findings and immunohistochemistry. Histopathological examination of the resected PRNET. H&E staining: In hematoxylin and eosin staining, a well-circumscribed infiltration by an invasive epithelial neoplasm is observed, exhibiting tubular, trabecular, and papillary architectural arrangements. The neoplastic cells display polygonal to tall columnar morphology with abundant eosinophilic cytoplasm and centrally located round-to-oval nuclei containing finely granular chromatin. Lymphocytic infiltrates are present predominantly at the tumor margin and occasionally within the central areas of the lesion (magnification, 20× main image, 40× section). Synaptophysin staining: tumor infiltrates are positive for synaptophysin (magnification 20×). Chromogranin A staining: chromogranin A shows cytoplasmic positivity (magnification 20×). CA9 staining: the tumor infiltrates are positive for carbonic anhydrase 9 (magnification 20×). Ki67 staining: the cell proliferation rate was very low with Ki67 positivity in 1.6% of tumor cells (digital assessment) (magnification 20×). SSTR2 staining: 60% of the tumor cells exhibit moderate to strong positivity for SSTR2 (magnification 20×). Islet-1 staining: the tumor infiltrates are positive for Islet-1 (magnification 20×). OTP staining: OTP staining is negative in the tumor infiltrates (magnification 20×). TTF-1 staining: the tumor infiltrates are negative for TTF-1 (magnification 20×). Abbreviations: CA9 = carbonic anhydrase 9, H&E = hematoxylin and eosin, OTP = orthopedia homeobox protein, PRNET = primary renal neuroendocrine tumor, SSTR2 = somatostatin receptor 2, TTF-1 = thyroid transcription factor 1.

**Figure 3:**
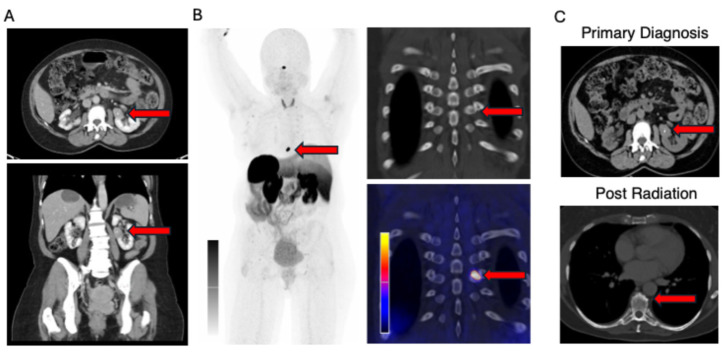
Radiological findings. (**A**) Initial preoperative CT scan showing a left-sided renal tumor (red arrows). (**B**) Postoperative SSTR PET/CT revealing a suspected metastatic lesion in thoracic vertebra 8 (T8) with high SSTR expression (red arrows). (**C**) Additional CT scan showing the left-sided renal tumor at first diagnosis (red arrows) and metastatic lesion at thoracic vertebra 8 (T8) post radiation (red arrow). Abbreviations: CT = Computed Tomography, SSTR PET/CT = Somatostatin Receptor Positron Emission Tomography/Computed Tomography.

SSTR2 immunohistochemistry demonstrated moderate receptor expression (SSTR2 Score: 3+ (with more than 50% of tumor cells showing a membranous staining)), supporting the neuroendocrine nature of the tumor and correlating with functional imaging findings. Collectively, the morphological and immunophenotypic features supported the diagnosis of a well-differentiated primary renal neuroendocrine tumor (PRNET, G1) (Figure 2). A follow-up SSTR PET/CT scan, performed in January 2025, showed a suspected metastatic lesion in thoracic vertebra 8 (T8) with high SSTR expression (Figure 3). Since fractionated radiotherapy of the osseous metastatic lesion at T8 represents standard of care for spinal bone metastases, in accordance with the tumor board recommendation, a fractionated irradiation of the metastatic lesion T8, using 25 Gy (5 Gy × 5 times), was performed between February and March 2025. Recent imaging performed in June 2025 showed a stable size of the lesion in T8 and no evidence of other lesions.

In order to enhance the understanding of this exceedingly rare tumor entity, a comprehensive analysis of the tumor tissue was conducted via whole-exome sequencing and extended transcriptome analysis. For the exome sequencing, a Somatic Cancer panel (target region: VirtualTumorPanel_v5_exon20, bases: 4634576, and genes: 1160) was used. The tumor content in the analyzed tissue sample was 70%. The tumor mutational burden was 1 variant per megabase (Var/Mb). No evidence of microsatellite instability was found. Neither fusions nor structural variants were detected. Virus DNA was also not detected. With a homologous recombination deficiency (HRD) score of 0, there were no indications of HRD. The copy number variation (CNV) burden was 9%. Furthermore, the following potentially relevant somatic alterations were identified: heterozygous deletion of BAP1, heterozygous deletion of FANCD2, heterozygous deletion of MLH1, heterozygous deletion of SMAD4, heterozygous deletion of TP53, heterozygous deletion of VHL, and non-focal amplification of TERT. To assess a potential inactivation of the second allele of these genes through methylation, immunohistochemical staining was performed; however, no loss of expression was confirmed. For the transcriptome analysis, the whole RNA was transcripted into cDNA. PolyA-cDNA was prepared for sequencing using New England Biolabs NEBNext Ultra II Directional RNA Library Prep. Afterwards, paired-end reads were sequenced using NovaSeq6000 (Novogene Co., Ltd. (Beijing, China)). No oncogenic structural alterations were identified. Owing to the absence of appropriate reference samples, differential expression analysis could not be conducted. Nevertheless, increased expressions of FGFR1, JUN, and SYK, as well as reduced expressions of CDKN2A, CDKN2B, and CDKN2C, were observed. In summary, no relevant renal cell cancer-related gene alterations (e.g., VHL, MET, FLCN, FH, BAP1, and SMARCA4) nor NEN-related gene alterations (e.g., ATRX, DAXX, MEN1, and TSC1/2) were detected.

## 3. Discussion

PRNETs are exceptionally rare entities [4]. The origin of PRNET remains a subject of ongoing debate, as neuroendocrine cells are not described as kidney-resident [5]. Hypotheses regarding their origin, aside from the possibility of metastases of an unknown primary tumor [7,8], include: metaplasia due to chronic inflammation [7,9], differentiation from neural crest cells, trapped during embryonic development [7,10], and activation of shared genetic sequences for neuroendocrine pathways in pluripotent primitive stem cells [7,11].

Compared to tumors from other primary organs, such as the digestive system [12,13] and the lungs [12,14], PRNETs are often large at the time of primary diagnosis, reflecting their retroperitoneal location and lack of initial symptoms. As a result, PRNETs at primary diagnosis show distant metastasis in 73% of cases [12,15]. In contrast to neuroendocrine tumors from other primary organs, PRNETs are infrequently associated with paraneoplastic syndromes, including Cushing syndrome and carcinoid syndrome [12,16].

The asymptomatic presentation remains a main challenge in the diagnosis and management of these tumors. Conventional imaging modalities, including CT and magnetic resonance imaging (MRI), often reveal a renal mass but fail to distinguish between the more prevalent renal cell carcinoma (RCC) and the rare PRNET. In line with RCC guidelines, surgical treatment in the form of nephrectomy or partial nephrectomy is frequently performed, and only after initial surgical resection, when pathological findings guide further investigation, functional imaging such as SSTR PET/CT is performed.

The pathological architecture of PRNET is described as trabecular with centrally located round-to-oval nuclei with granular eosinophilic cytoplasm and fine chromatin [12]. Chromogranin A as well as Islet-1 positivity are common in PRNET [6,12]. Islet-1 (ISL1, also known as Insulin gene enhancer protein 1), a transcription factor that plays a role in the embryonic development and differentiation of pancreatic β-cells in all endocrine cells, but not in exocrine cells [17,18], is known to be differentially expressed among various tumors, including pancreatic NET, duodenal NET, lower gastrointestinal neuroendocrine tumors, pulmonary neuroendocrine tumors, and middle ear NET [17]. However, Ilset-1 is not a primary marker for RCC. Therefore, it helps to distinguish between RCC and the likely Islet-1 positive PRNET [17].

Because of its ability to express pancreatic hormones, as well as its Islet-1 positivity, PRNET was supposed to be genetically similar to NET of pancreatic origin. In line with the findings of Kasajima et al. [12] and Pivovarcikova et al. [19], this case was negative for mutations in NET-related genes such as ATRX, DAXX, MEN1, and TSC 1/2, as well as negative for NEC-related genes such as TP53, RB1, and PIK3CA. BAP1 (BRCA1-associated protein-1), a tumor suppressor gene associated with an aggressive form of clear cell RCC [20,21], showed a heterozygous deletion in this case of a PRNET; nevertheless, the second allele was not inactivated. Moreover, SMARCA4, a core component of the SWI/SNF chromatin remodeling complex and therefore essential for the regulation of gene expression, is known to be frequently mutated in clear cell RCC [22]. In our case of a PRNET, SMARCA4 showed no alterations. The most common gene alterations in clear cell RCC affect the tumor suppressor gene VHL (von Hippel-Lindau) [23,24]. In this PRNET case, a somatic heterozygous deletion of VHL was detected, but immunohistochemical expression was not decreased. According to these findings, in this case of a PRNET, no relevant renal cell cancer-related gene alterations nor NEN-related gene alterations were detected, leading to the hypothesis that the genomic landscape in PRNET may be unique.

Due to the infrequency of PRNET, there are currently no standardized, evidence-based treatment guidelines. Complete surgical resection remains the cornerstone of treatment, particularly in a non-metastasized disease. In alignment with the treatment protocols for other neuroendocrine tumors, somatostatin analogs (SSA) are frequently used for well-differentiated, somatostatin receptor-positive tumors to manage symptoms and inhibit tumor progression. Additionally, therapeutic approaches such as peptide receptor radionuclide therapy (PRRT), targeted therapies like everolimus (an mTOR inhibitor), and even platinum-based chemotherapy regimens (e.g., cisplatin/etoposide) may be considered for more aggressive tumor types. Prognosis varies significantly and has recently been demonstrated to be highly dependent upon tumor size (5-year progression-free survival (PFS) rate of PRNET is 65% for tumors <6 cm and 31% for tumors ≥6 cm) and the presence of metastasis [12].

This case underscores not only the diagnostic challenges associated with primary renal neuroendocrine tumors (PRNETs) but also highlights the critical necessity for ongoing research into this rare entity, particularly concerning the elucidation of the genetic signature of PRNETs to clearly distinguish them from more frequent RCC. Accurate categorization is crucial for adherence to treatment guidelines, as the biological behavior and optimal treatment strategies for PRNET differ from those for other renal cancers. Understanding the unique characteristics of these rare tumors is essential for appropriate clinical decision-making and improving patient outcomes.

## Data Availability

Due to privacy or ethical restrictions, the data presented in this study are only available upon specific request.
